# Correlates of Physical Activity for Adults With Disability

**Published:** 2006-06-15

**Authors:** Sarah E Boslaugh, Elena M Andresen

**Affiliations:** Department of Pediatrics, Washington University School of Medicine; At the time of the study, Dr Boslaugh was with the Health Communications Research Laboratory at the Saint Louis University School of Public Health; Epidemiology Division, College of Public Health and Health Professions, University of Florida Health Science Center, Gainesville, Fla; At the time of the study, Dr Andresen was with the Department of Community Health at the Saint Louis University School of Public Health

## Abstract

**Introduction:**

This study was designed to determine factors that influence the physical activity level of adults with disability as identified in a large representative sample of U.S. adults.

**Methods:**

Data were taken from the District of Columbia and the 12 states that administered the Quality of Life and Caregiving Module of the 2001 Behavioral Risk Factor Surveillance System. Adults with disability (n = 4038) were defined as those who required special equipment because of a health problem or who required the assistance of another person either for their personal care or routine needs. Adequate physical activity was defined as meeting the Centers for Disease Control and Prevention and American College of Sports Medicine recommendation of at least 30 minutes of moderate activity per day at least 5 days per week. Unadjusted and adjusted odds ratios were computed for demographic, health status, health care access, and health behavior variables.

**Results:**

Only one fourth of the study population met the recommendation for moderate activity level. African American race, age of 50 years or older, annual income of $50,000 or higher, and being in good, fair, or poor health were all significantly related to activity level; sex, education level, health care access, and years of disability were not.

**Conclusion:**

Adults with disability are not meeting basic recommendations for physical activity. Some correlates of physical activity found in general populations are also related to activity level for people with disability (age, general health, race), whereas others (sex, education level) are not. These factors should be considered when planning physical activity interventions for people with disability.

## Introduction

Approximately 200,000 to 300,000 premature deaths occur each year in the United States because of physical inactivity (1-4). Despite the benefits of regular activity, only 31% of adults in the United States report engaging in recommended amounts of physical activity (i.e., 30 minutes of moderate-intensity activity 5 or more days per week or 20 minutes of vigorous-intensity activity 3 or more days per week), and 38% report no leisure-time regular physical activity (5). Activity levels are even lower among people with disability; for example, *Healthy People 2010* reports that 56% of people with disability reported no leisure-time physical activity, compared with 36% of people without disability, and rates of participation in regular moderate and vigorous physical activity are also lower for people with disability (6). This is particularly important because physical activity is similarly beneficial for people with or without disability and has been shown to improve quality of life and reduce functional impairment among people with disability (7-14). Accordingly, the goal of increasing physical activity is one of 10 leading indicator areas within *Healthy People 2010*, and people with disability are specifically included within the target population (15).

Correlates of physical activity among adults without disability are well-known and consistent across many studies; they include sex, age, income, race, education, obesity, and general health status. There has been less research on the correlates of physical activity for adults with disability. Disability has often been included as one item on a list of barriers to physical activity, often addressed within an item asking about injury or disability or as a barrier or correlate, such as arthritis, obesity, and asthma (16-19). There have also been studies on activity levels of people with medical conditions that can be disabling, such as arthritis (20).

Recently, a few researchers have begun to study the correlates of activity level for people with disability. For instance, Simonsick et al examined walking activity in a group of elderly women with moderate to severe disability and found that even when degree of disability was considered, race, psychosocial factors, and specific impairments remained significant predictors of activity level (21). Kinne et al found that barriers, motivation, and self-efficacy were predictors of exercise maintenance in a group of people with mobility impairments, but demographic factors were not (22). Shifflett et al found that perceived benefits, facilities barriers, and health barriers were important predictors of activity level in people with disability (23). Rimmer et al identified several barriers to physical activity for people with disability, including cost, lack of transportation, and inaccessibility (24-26). Two instruments for measuring activity level for people with disability have also been reported (27,28), indicating interest in evaluating physical activity in this population. However, no studies have examined large samples of people with broadly defined classes of disability for the purpose of finding correlates of physical activity that may be used to construct large-scale interventions for people with disability.

## Methods

### Sample

Data analyzed for this study were taken from the 2001 Behavioral Risk Factor Surveillance System (BRFSS) database (29-31). The BRFSS is an annual random-digit–dialed telephone survey of noninstitutionalized U.S. adults (aged 18 or older). The BRFSS consists of core questions, which are asked in all states, and modules that individual states may elect to use or not. Because several questions used in this study came from the optional Quality of Life and Caregiving Module, only data from the District of Columbia and the 12 states that administered that module in 2001 are included in this study: Alaska, Arizona, Delaware, Georgia, Maryland, Minnesota, Nebraska, New Jersey, Ohio, Tennessee, Utah, and Virginia. The project was approved by the Saint Louis University Institutional Review Board.

### Variables

**Physical activity**

The outcome of interest is physical activity level. For comparability with other studies, the recommendation of the Centers for Disease Control and Prevention (CDC) and American College of Sports Medicine (ACSM) for moderate physical activity was used: an adult performing moderate exercise for at least 30 minutes on 5 or more days per week in segments of at least 10 minutes each is considered to be sufficiently active (32). Participants were classified dichotomously as meeting this recommendation or not.

**Disability**

Measurement of disability is problematic because several competing models of disability and different classification systems have been used in different studies (33-37). For this study, we adapted an approach previously used with BRFSS data (38), in which adults giving positive responses to either of two core questions are classified as having a disability:

1. Are you limited in any way in any activities because of any impairment or health problem?
2. Do you now have any health problem that requires you to use special equipment, such as a cane, wheelchair, special bed, or special telephone?

We augmented this approach by combining it with responses to two questions from the Quality of Life and Caregiving Module:

1. Because of any impairment or health problem, do you need the help of other persons with your personal care needs, such as eating, bathing, dressing, or getting around the house?
2. Because of any impairment or health problem, do you need the help of other persons in handling your routine needs, such as everyday household chores, doing necessary business, shopping, or getting around for other purposes?

We classified people who answered yes to questions 2, 3, or 4 as having a disability. Therefore, people who indicated that they required special equipment because of a health problem or who required the assistance of another person either for their personal care or routine needs were classified as having disability. Adults who answered yes only to question 1 or to none of the questions were classified as not having disability. The analysis began with 47,179 cases; 807 cases did not have sufficient information to classify disability status and were removed from the analysis. Of the remaining 46,372 cases (98.3%), 4038 (8.7%) were classified as having disability and 42,334 as not having disability.

### Correlates

Because the purpose of this study was primarily exploratory (i.e., to find correlates of physical activity among people in broadly defined classifications of disability), numerous independent variables were included as potential correlates. Seven demographic variables were included: age, race and ethnicity, sex, education level, employment, income, and marital status. Age was categorized in years as 18 to 29, 30 to 49, 50 to 64, 65 to 79, and 80 and older. Race and ethnicity was categorized as white non-Hispanic, African American non-Hispanic, other non-Hispanic, and Hispanic. Education level was categorized as less than high school, high school graduate, some college, and college graduate. Employment was categorized as working or homemaker, not working, student, or unable to work. Income was classified into eight categories, from less than $10,000 to $75,000 or more annually. Marital status was categorized as married, divorced, widowed, separated, never married, or member of an unmarried couple. Adults were also classified by whether they lived alone or with other people. Access to health care was measured by two dichotomous variables: having health plan coverage and having a personal doctor. Two health status variables were included: general health status (excellent, very good, good, fair, and poor) and body mass index (underweight, normal weight, overweight, or obese). Three chronic disease variables were included: currently have asthma, have a medical diagnosis of diabetes, and have a medical diagnosis of arthritis. Years of disability was categorized as 0 to 1 year, 2 to 4 years, 5 to 9 years, and 10 or more years. Four variables classified smoking and drinking behavior: current smoking, lifetime smoking (have smoked 100 cigarettes in lifetime), current alcohol consumption (none, moderate, or heavy), and binge drinking.

### Statistical analysis

We conducted two sets of analyses using SPSS 11.0 for Macintosh System X (SPSS Inc, Chicago, Ill). First, using *t* tests and chi-square tests, we compared the samples with and without disability on numerous factors. Second, we used logistic regression on the sample of adults with disability to compute the unadjusted and adjusted odds ratios (ORs) and 95% confidence intervals (CIs) for potential correlates of physical activity level.

## Results

Table 1 presents results of the four BRFSS disability classification questions for the entire study population (n = 46,372) as well as results of the questions on major impairment or health problem and length of disability for people who answered yes to either question 1 or question 2 (n = 8348). Responses are provided by sex and two age categories (younger than 65 or 65 and older). Results are also presented for combinations of BRFSS disability classification questions. Chi-square tests were used to test differences in responses between men and women and people younger and older than age 65 on the disability questions. Adults aged 65 or older were significantly more likely to answer yes to three of the four disability classification questions (not to "need help with personal care") and to multiple questions (indicating a higher level of disability). Women were significantly more likely than men to answer affirmatively to the disability questions. Major impairment or health problem differed by age: adults 65 and older were most likely to report arthritis (26.5%) followed by a walking problem (11.5%), whereas adults under 65 were most likely to report back or neck problems (18.5%), followed by arthritis/rheumatism (13.5%). Women were most likely to report arthritis/rheumatism as their chief problem, followed by back or neck problems, whereas men were most likely to report back or neck problems (17.2%), followed by fractures or bone or joint injury (12.4%). Men were significantly more likely than women to have had disability for 10 years or longer, as were people 65 years and older compared with people younger than 65 years.

Table 2 presents descriptive characteristics of the study population with disability and without disability; chi-square tests were used to test the differences between the samples with and without disability. All group differences except race were significant at the .05 level. About half as many adults with disability met the moderate activity standard (25.4%) as adults without disability (43.3%). Adults with disability had lower incomes and less education and were older, more likely to be female, less likely to be married, more likely to live alone, and less likely to be employed than adults without disability. They were also in worse health; more likely to have diabetes, arthritis, or asthma; and more likely to be obese.

Unadjusted and adjusted ORs for each correlate are presented in Table 3. The unadjusted ORs show relationships similar to those found in studies of the general population. For adults with disability, male sex, increasing age, higher educational level and income, and good health were all associated with increased likelihood of meeting the moderate activity standard. African American race was negatively associated with activity level, as was obesity and having diabetes or arthritis. Alcohol consumption was positively related to activity level, but smoking did not have a significant relationship with activity. Being unable to work had a strong negative relationship with activity level, as did not currently working.

After adjustment for other correlates, only a few variables remain significant predictors of activity level. Increasing age is negatively related to activity level. African American race is negatively associated with sufficient activity level. Only the two highest income categories ($50,000–$74,999 and ≥$75,000) are significant predictors of activity compared to the lowest category (<$10,000). The only employment category that was a significant predictor was being unable to work compared with working. The three lowest categories of general health (good, fair, and poor) are significant predictors of insufficient activity level compared with the highest category (excellent). Of the three chronic medical conditions included, only asthma is a significant predictor, and paradoxically it is positively associated with sufficient activity.

## Discussion

Adults with disability in the United States are not achieving activity levels recommended by CDC and ACSM, and in fact only about half as many meet the recommendations for moderate activity compared with adults without disability. This suggests that a special effort should be made to encourage physical activity among people with disability. Any effort to develop interventions for people with disability must be based on knowledge of correlates of physical activity for that population. This study is the first to investigate correlates of physical activity in a large sample of people from numerous geographical regions using a broad definition of disability.

Some of the correlates of physical activity for the population with disability found in this study are similar to correlates found in studies of the general population. Among these are African American race (negatively associated with physical activity), higher income (positively associated with activity), older age (negatively associated with activity), and poor general health (negatively associated with activity). Other correlates that we expected to be important (because they have been found to be related to activity levels in studies of the general population) were not significant predictors after adjustment for other covariates. These correlates include years of activity limitation, body mass index, education level, having diabetes or arthritis, and smoking behavior. Surprisingly, years of activity limitation was not related to physical activity level after controlling for other covariates.

The definition of disability used in this study is based on functional status rather than diagnosis of disease or medical condition. There are many ways to define and measure disability, and no definition is perfect; however, we believe that the definition we selected is appropriate for large-scale survey instruments that are administered to the general population and that must obtain disability information by using only a few questions. The combination of questions used in this study yields a broad classification, and people classified as having disability by these questions will certainly be heterogeneous on medical condition and personal limitations. However, national efforts to increase physical activity among people with disability cannot be designed to target separately each and every type and degree of disability but must use broad categories and common correlates in planning interventions. Analyses of large-sample surveys such as the BRFSS are an important part of identifying these correlates.

This study has several limitations. One is that some people with disability are excluded by design because the BRFSS only surveys the noninstitutionalized population and requires that individuals have a telephone and be willing and able to answer the survey questions. A second limitation is the broad classification of disability and the subjective questions used to make the classification: two people could have similar levels of impairment or disability by medical or legal definition and yet answer the classification questions differently. However, the current definition of disability is applied as a social and demographic descriptor and not a medical or legal definition, so these self-definitions are appropriate for this purpose. In addition, the questions are the product of extensive national discussions and constituent feedback, and the first two questions (the first on limitations and the second on the requirement of special equipment) are also used in the National Health and Nutrition Examination Survey and the National Health Interview Survey (39). A third limitation is that the 2001 BRFSS did not include questions about some topics that have been shown to be strongly related to exercise in people with disability; these include barriers such as cost (24) and inaccessible built environments (25,26) as well as social support and psychological factors such as perceived competence and perceived benefits of exercise and exercise self-efficacy (22,23). A fourth limitation is that the data were drawn from only the District of Columbia and the 12 states that administered the Quality of Life and Caregiving Module of the BRFSS in 2001. However, we have no reason to suspect that these relationships would vary if all state BRFSS respondents were represented.

## Figures and Tables

**Table 1:** Responses to Disability Questions (n = 46,372), Behavorial Risk Factor Surveillance System, 2001^a^

**Question**	**Men, %**	**Women, %**	***P *Value**	**Age <65 y, %**	**Age ≥65 y, %**	***P *Value**	**Total, %**
**Behavioral Risk Factor Surveillance System, 2001^b^**							
Yes to question 1: limited in activities	15.5	17.0	<.001	14.5	25.2	<.001	16.4
Yes to question 2: need to use special equipment	5.3	6.3	<.001	4.0	14.9	<.001	5.9
Yes to question 3: need help with personal care	1.4	1.9	<.001	1.5	2.7	<.001	1.7
Yes to question 4: need help with routine needs	3.2	6.7	<.001	4.3	9.9	<.001	5.3
Yes to both questions 1 and 2	2.6	4.6	<.001	3.0	10.1	<.001	4.2
Yes to both questions 1 and 3	1.3	1.8	<.001	1.4	2.4	<.001	1.6
Yes to both questions 1 and 4	2.9	6.3	<.001	4.2	8.4	<.001	4.9
Yes to both questions 2 and 3	0.9	1.2	.01	0.8	2.1	<.001	1.0
Yes to both questions 2 and 4	1.8	3.2	<.001	1.7	6.8	<.001	2.6
Yes to both questions 3 and 4	1.0	1.7	<.001	1.2	2.2	<.001	1.4
Yes to questions 2, 3, or 4	6.8	10.0	<.001	6.7	18.1	<.001	8.7
**What is your major impairment or health problem?^c^^,^^d^**			<.001			<.001	—
Arthritis/rheumatism	11.8	20.7	—	13.5	26.5	—	16.8
Back or neck problems	17.2	14.2	—	18.5	7.6	—	14.9
Fractures, bone/joint injury	12.4	8.4	—	11.2	6.8	—	9.6
Depression/anxiety/emotional problem	5.4	6.7	—	8.3	1.5	—	6.0
Walking problem	5.1	6.8	—	4.0	11.5	—	6.0
Other	48.2	43.1	—	44.5	46.2	—	45.0
**For how long have your activities been limited because of your major impairment or health problem?^d^**			<.001			<.001	—
0-1 y	26.1	26.9	—	27.8	23.4	—	26.6
2-4 y	22.3	26.5	—	23.9	27.4	—	24.9
5-9 y	19.2	20.4	—	20.0	19.9	—	20.0
≥10 y	32.4	26.1	—	28.3	29.2	—	28.5

a Data were used from the District of Columbia and the 12 states that administered the Quality of Life and Caregiving Module: Alaska, Arizona, Delaware, Georgia, Maryland, Minnesota, Nebraska, New Jersey, Ohio, Tennessee, Utah, and Virginia.

b Question 1 addressed limitation in activities because of impairment or health problem; question 2, problems that require special equipment, such as a cane, wheelchair, special bed, or special telephone; question 3, needing help of others with personal care needs, such as eating, bathing, dressing, or getting around house; question 4, needing help of others with routine needs, such as everyday household chores, doing necessary business, shopping, or getting around for other purposes.

c Table includes only the top overall five responses.

d Questions on major impairment and years of disability were only asked of people who answered yes to BRFSS questions 1 and 2 (n = 8348).

**Table 2:** Characteristics of Adults Without Disability (n = 46,372) and Adults With Disability (n = 4038), Behavioral Risk Factor Surveillance System, 2001^a^

**Characteristic**	**Adults Without Disability,** **%**	**Adults With Disability,** **%**	***P *Value**
**Meets moderate activity standard recommended by CDC and ACSM^b^**	43.3	25.4	<.001
**Age, y**			<.001
18-29	19.0	5.8	—
30-49	43.2	29.6	—
50-64	21.9	27.7	—
65-79	13.1	24.5	—
≥80	2.8	12.4	—
**Race and ethnicity**			.06
White non-Hispanic	76.8	77.5	—
African American non-Hispanic	12.2	12.7	—
Other non-Hispanic	5.2	4.4	—
Hispanic	5.9	5.3	—
**Male sex**	41.4	31.7	<.001
**Education level**			<.001
Less than high school	9.0	21.6	—
High school graduate	30.7	32.7	—
Some college	27.4	25.7	—
College graduate	33.0	19.9	—
**Employment**			<.001
Working or homemaker	75.5	32.5	—
Not working	20.0	40.7	—
Student	3.0	1.0	—
Unable to work	1.6	25.8	—
**Annual income, $**			<.001
<10,000	3.8	15.4	—
10,000-14,999	4.2	11.8	—
15,000-19,999	6.9	13.6	—
20,000-24,999	9.0	12.6	—
25,000-34,999	15.2	13.5	—
35,000-49,999	19.5	12.9	—
50,000-74,999	18.4	9.9	—
≥75,000	22.8	10.3	—
**Marital status**			<.001
Married	54.5	42.8	—
Divorced	12.8	16.8	—
Widowed	8.2	22.8	—
Separated	2.7	3.8	—
Never married	19.1	12.0	—
Member of an unmarried couple	2.7	1.9	—
**Live alone**	32.2	46.2	<.001
**Health care access**			
Have health insurance	88.7	90.4	.001
Have personal doctor	80.5	89.5	<.001
**General health**			<.001
Excellent	24.4	5.4	—
Very good	36.6	12.8	—
Good	28.4	25.5	—
Fair	8.7	28.9	—
Poor	1.9	27.3	—
**Chronic medical conditions**			
Diabetes	5.4	17.8	<.001
Arthritis	20.3	59.6	<.001
Asthma	6.1	17.3	<.001
**Body mass index**			<.001
Underweight (<18.5)	1.9	3.1	—
Normal (18.5-24.9)	42.1	31.4	—
Overweight (25-29.9)	36.6	32.0	—
Obese (≥30)	19.4	33.5	—
**Smoking**			
Current smoker	21.9	25.9	<.001
Has smoked 100 cigarettes in lifetime	45.7	57.5	<.001
**Alcohol consumption: type of drinker**			<.001
Do not drink alcohol	46.7	67.5	—
Moderate drinker	48.4	29.6	—
Heavy drinker	5.0	2.9	—
At risk for binge drinking	13.1	6.3	<.001

a Data were used from the District of Columbia and the 12 states that administered the Quality of Life and Caregiving Module: Alaska, Arizona, Delaware, Georgia, Maryland, Minnesota, Nebraska, New Jersey, Ohio, Tennessee, Utah, and Virginia.

b The Centers for Disease Control and Prevention (CDC) and American College of Sports Medicine (ACSM) recommend 30 minutes of moderate-intensity physical activity on at least 5 days per week in segments of at least 10 minutes (32).

**Table 3:** Unadjusted and Adjusted Odds Ratios for Adequate Physical Activity Level^a^ for Adults With Disability (n = 4038), Behavioral Risk Factor Surveillance System, 2001^b^

**Characteristic**	**Unadjusted OR(95% CI)**	**Adjusted OR(95% CI)**
**Age, y**		
18-29	Ref	Ref
30-49	0.53 (0.40-0.71)	0.76 (0.51-1.14)
50-64	0.36 (0.27-0.49)	0.61 (0.39-0.95)
65-79	0.24 (0.18-0.33)	0.54 (0.32-0.89)
≥80	0.18 (0.13-0.27)	0.40 (0.22-0.74)
**Race and ethnicity**		
White non-Hispanic	Ref	Ref
African American non-Hispanic	0.57 (0.44-0.73)	0.61 (0.44-0.84)
Other non-Hispanic	1.43 (1.03-1.97)	1.08 (0.69-1.69)
Hispanic	1.12 (0.82-1.52)	1.06 (0.70-1.61)
**Sex**		
Female	Ref	Ref
Male	1.36 (1.17-1.58)	1.17 (0.95-1.44)
**Education level**		
Less than high school	Ref	Ref
High school graduate	1.87 (1.50-2.34)	1.42 (1.06-1.91)
Some college	2.05 (1.63-2.58)	1.23 (0.90-1.68)
College graduate	3.00 (2.35-3.77)	1.36 (0.97-1.92)
**Employment**		
Working or homemaker	Ref	Ref
Not working	0.38 (0.32-0.44)	0.78 (0.60-1.03)
Student	1.21 (0.64-2.30)	0.75 (0.34-1.64)
Unable to work	0.32 (0.27-0.39)	0.65 (0.49-0.85)
**Annual income, $**		
<10,000	Ref	Ref
10,000-14,999	1.20 (0.83-1.64)	1.05 (0.71-1.54)
15,000-19,999	1.30 (0.94-1.79)	1.02 (0.70-1.47)
20,000-24,999	1.47 (1.06-2.02)	1.00 (0.68-1.46)
25,000-34,999	1.62 (1.19-2.22)	1.06 (0.72-1.56)
35,000-49,999	2.58 (1.90-3.49)	1.38 (0.93-2.04)
50,000-74,999	2.90 (2.10-4.00)	1.61 (1.05-2.47)
≥75,000	3.78 (2.61-4.90)	1.88 (1.21-2.91)
**Marital status**		
Married	Ref	Ref
Divorced	0.79 (0.64-0.97)	1.13 (0.83-1.55)
Widowed	0.46 (0.38-0.57)	0.78 (0.54-1.14)
Separated	0.89 (0.61-1.29)	1.38 (0.84-2.73)
Never married	1.08 (0.87-1.35)	1.13 (0.79-1.60)
Member of unmarried couple	1.85 (1.16-2.96)	2.35 (1.26-4.40)
**Living arrangement**		
Lives with other(s)	Ref	Ref
Lives alone	0.74 (0.64-0.85)	1.03 (0.86-1.42)
**Health insurance**		
Has health insurance	Ref	Ref
No health insurance	1.26 (1.00-1.58)	1.09 (0.79-1.50)
**Personal doctor**		
Has personal doctor	Ref	Ref
No personal doctor	1.80 (1.46-2.23)	1.26 (0.94-1.70)
**General health**		
Excellent	Ref	Ref
Very good	0.71 (0.51-0.97)	0.95 (0.62-1.45)
Good	0.46 (0.34-0.62)	0.64 (0.42-0.95)
Fair	0.34 (0.25-0.46)	0.56 (0.37-0.84)
Poor	0.19 (0.14-0.26)	0.35 (0.23-0.54)
**Chronic medical conditions**		
Absence of the condition	Ref	Ref
Diabetes	0.63 (0.51-0.77)	1.11 (0.85-1.44)
Arthritis	0.58 (0.50-0.67)	1.08 (0.89-1.32)
Asthma	1.61 (0.97-1.40)	1.32 (1.04-1.67)
**Years of activity limitation**		
0-1	Ref	Ref
2-4	0.64 (0.52-0.79)	0.83 (0.64-1.06)
5-9	0.79 (0.63-0.98)	0.90 (0.69-1.18)
≥10	0.83 (0.68-1.01)	0.88 (0.69-1.12)
**Body mass index**		
Underweight (<18.5)	0.77 (0.50-1.20)	1.00 (0.58-1.76)
Normal (18.5-24.9)	Ref	Ref
Overweight (25.0-29.9)	0.84 (0.70-1.00)	0.81 (0.46-1.43)
Obese (≥30)	0.64 (0.53-0.77)	0.64 (0.36-1.12)
**Smoking**		
Not a current smoker	Ref	Ref
Current smoker	1.17 (1.00-1.37)	0.80 (0.62-1.03)
Has smoked fewer than 100 cigarettes in lifetime	Ref	Ref
Has smoked 100 cigarettes in lifetime	1.16 (1.00-1.33)	0.84 (0.68-1.05)
**Alcohol consumption**		
Does not drink alcohol	Ref	Ref
Moderate drinker	1.85 (1.59-2.16)	1.01 (0.82-1.25)
Heavy drinker	2.52 (1.72-3.69)	1.54 (0.91-2.62)
At risk for binge drinking	1.97 (1.51-2.56)	0.93 (0.63-1.38)

OR indicates odds ratio; CI, confidence interval; ref, reference group.

a The Centers for Disease Control and Prevention (CDC) and American College of Sports Medicine (ACSM) recommend 30 minutes of moderate-intensity physical activity on at least 5 days per week.

b Data were used from the District of Columbia and the 12 states that administered the Quality of Life and Caregiving Module: Alaska, Arizona, Delaware, Georgia, Maryland, Minnesota, Nebraska, New Jersey, Ohio, Tennessee, Utah and Virginia.
